# Between reality and meaning: a qualitative study on virtual reality as a compassionate technology in end-of-life care

**DOI:** 10.1080/17482631.2026.2669695

**Published:** 2026-05-06

**Authors:** Manaporn Chatchumni, Petpailin Phibunnithikasem, Ravinan Thatsiriniratkul, Thongdech Prasertsri, Rawiphon Chotikunnan, Phichitphon Chotikunnan

**Affiliations:** a School of Nursing, Rangsit University, Pathumthani, Thailand; b Helth Welfare office, Rangsit University, Pathumthani, Thailand; c Maha Vajiralongkorn Thanyaburi Hospital, Pathumthani, Thailand; d College of Biomedical Engineering, Rangsit University, Pathum Thani, Thailand

**Keywords:** Virtual reality, palliative care, end-of-life care, spirituality, qualitative research, Thailand

## Abstract

**Background:**

Virtual reality (VR) is increasingly recognized as a supportive tool in palliative care due to its potential to promote relaxation, emotional comfort, and spiritual reflection. In Thailand, where end-of-life care is shaped by family relationships, Buddhist beliefs, and the value of a peaceful death, little is known about how VR is perceived in this context.

**Aim:**

To explore perceptions, emotional and spiritual needs, and anticipated acceptability of VR in end-of-life care among terminally ill patients, family caregivers, and palliative care professionals in Thailand.

**Method:**

A qualitative descriptive study was conducted using semi-structured interviews with 15 purposively selected participants, including five terminally ill patients, five family caregivers, and five healthcare professionals. The study focused on anticipated rather than directly experienced use of VR. Interviews were conducted in Thai, audio-recorded, transcribed verbatim, and analyzed using inductive content analysis. Rigor was enhanced through investigator triangulation, reflexive memo writing, peer debriefing, and an audit trail.

**Results:**

Four interrelated themes were developed: (1) emotional and existential needs, including longing for home, peace, and unresolved concerns; (2) spirituality and meaning, where VR was perceived as a medium for reflection and religious connection; (3) facilitators and barriers, including perceived benefits alongside concerns about usability, emotional impact, and staff workload; and (4) compassionate innovation, reflecting the potential of VR to support dignity, comfort, and meaning at the end of life. Participants viewed VR not merely as a distraction tool, but as a means of symbolic connection and emotional support.

**Conclusion:**

In this Thai palliative care context, VR was perceived as a potentially compassionate and culturally meaningful technology that may support emotional, existential, and spiritual well-being when tailored to patient readiness, cultural values, and clinical feasibility. As findings reflect anticipated perceptions, further research is needed to evaluate its practical, emotional, and ethical impacts in clinical settings.

## Introduction

End-of-life care requires more than symptom control alone. For people living with terminal illness, quality of life is also shaped by emotional comfort, existential peace, dignity, and a sense of meaning as death approaches (National Health Commission Office, 2024). In response, palliative care has increasingly adopted a holistic orientation that attends not only to physical suffering, but also to psychological, social, and spiritual well-being. Within this broader approach, VR has gained attention as a potentially valuable supportive technology in end-of-life care.

VR is increasingly recognised as more than a recreational or entertainment tool. Because it can create a strong sense of presence and immersive engagement, VR may enable individuals with severe physical limitations to experience meaningful places, memories, or symbolic environments that would otherwise be inaccessible (Woo, 2025). In palliative care settings, previous studies suggest that VR may reduce distress, promote relaxation, and support emotional well-being by offering immersive experiences that foster comfort, distraction, reflection, and personal connection (Gaina et al., 2024; Maheta et al., 2025; Martin et al., 2022; Moscato et al., 2021). Emerging discussions have also begun to frame VR as a more human-centred or compassionate technology, particularly when it is used to preserve dignity, agency, and selfhood in the context of advanced illness (American Bar Association, 2024; Pohan et al., 2024).

In Thailand, end-of-life care is deeply shaped by sociocultural and spiritual values. Family relationships, Buddhist beliefs, and the cultural aspiration for a peaceful and dignified death influence how patients and families understand suffering, acceptance, and preparation for death (National Health Commission Office, 2024; Phetcharat et al., 2023). However, despite growing international interest in VR for palliative care, relatively little is known about how this technology is perceived in Thai contexts, particularly in relation to emotional needs, spiritual meaning, and cultural appropriateness at the end of life.

## Background

Research on VR in palliative care has primarily focused on its potential to relieve anxiety, improve mood, and provide temporary escape from physical or emotional distress (Gaina et al., 2024; Martin et al., 2022; Moscato et al., 2021). These findings suggest that immersive environments may offer comfort to patients whose mobility and life spacing have become increasingly restricted by advanced illnesses. Some studies also indicate that VR may support reminiscence, relaxation, and opportunities for symbolic experiences, such as revisiting meaningful places or engaging in calming natural or spiritual settings (Groninger, 2022; Martin et al., 2022).

At the same time, end-of-life suffering is not limited to physical symptoms. Patients commonly experience emotional distress, fear, loss of autonomy, unfinished relationships, and existential concerns about identity, meaning, and mortality (National Health Commission Office, 2024). In this context, technologies such as VR may have relevance not only for symptom distraction, but also for emotional release, spiritual reflection, and meaning making. This broader perspective is especially important in palliative care, where well-being is often closely tied to one’s sense of peace, dignity, and connection to others.

Spirituality is particularly significant in Thai end-of-life care. Thai cultural understandings of a “good death” are often intertwined with Buddhist concepts of acceptance, calmness, moral preparation, and relational harmony, while family members frequently play a central role in emotional support and decision-making (National Health Commission Office, 2024; Phetcharat et al., 2023; Tantalanukul et al., 2023). These factors suggest that the perceived value of VR in Thai palliative care may differ from that described in predominantly Western literature, where assumptions about individual autonomy, technological use, and spiritual expression may not fully reflect collectivist or non-Western experiences.

Although media accounts and emerging research have highlighted moving examples of VR use near the end of life, such as enabling patients to revisit meaningful places or experience symbolic journeys, evidence from non-Western settings remains limited (Groninger, 2022; KPIX | CBS News Bay Area, 2024; Martin et al., 2022). There is therefore a need to understand how terminally ill patients, family caregivers, and healthcare professionals in Thailand perceive the possible use of VR before implementation. Exploring anticipated acceptability is important because it can help identify emotional benefits, cultural meanings, practical concerns, and ethical risks that should inform future intervention design.

## Purpose

The purpose of this study was to explore how terminally ill patients, family caregivers, and palliative care professionals in Thailand perceived the potential use of virtual reality in end-of-life care, with particular attention to emotional needs, spiritual meaning, anticipated benefits, anticipated risks, and contextual feasibility.

## Research questions

1. How do participants describe emotional, existential, and spiritual needs at the end of life?
2. How do they perceive the possible role of VR in addressing those needs?
3. What benefits, concerns, and implementation conditions do they anticipate in Thai palliative care settings? (Figure 1).

## Conceptual synthesis of VR in care

**Figure 1.**
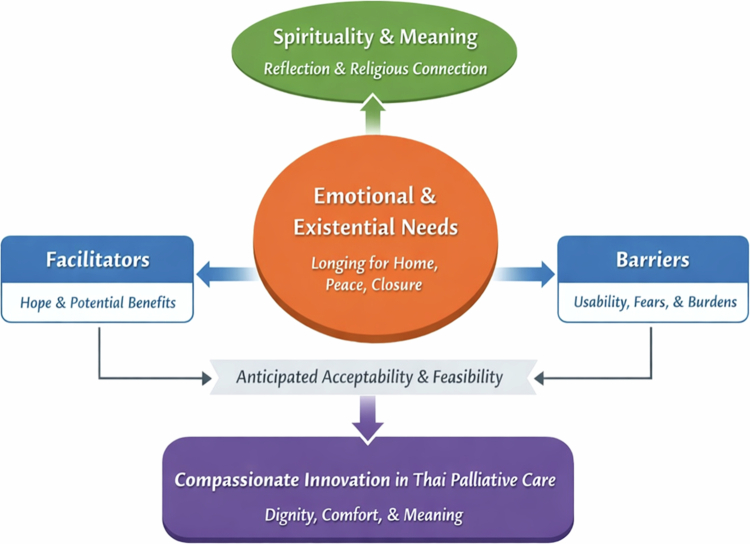
Conceptual synthesis of participants’ perceptions of virtual reality in end-of-life care. The figure illustrates how emotional and existential needs provide the central context for participants’ perceptions of VR, while spirituality and meaning shape its symbolic and reflective value. Facilitators and barriers influence anticipated acceptability and feasibility, and these dimensions together contribute to the broader concept of compassionate innovation in Thai palliative care.

## Material and methods

### Study design

This study used a qualitative descriptive design to explore perceptions, emotional and spiritual needs, and anticipated acceptability of VR in end-of-life care among terminally ill patients, family caregivers, and palliative care professionals. Qualitative description was selected because the study aimed to provide a comprehensive and practice-oriented account of participants’ perspectives on the potential role of VR in palliative care rather than to generate formal theory or examine the essential structure of lived experience, as in phenomenological research (Sandelowski, 2010; Sandelowski, 2000).

The study was informed by a constructivist interpretive stance, which recognises that experiences and meanings are shaped through sociocultural contexts and interpersonal interactions. From this perspective, participants’ accounts are understood as contextually situated interpretations of their experiences and expectations regarding end-of-life care and emerging supportive technologies. This approach was appropriate for exploring how VR may be perceived within the complex intersection of technology, spirituality, and holistic palliative care (Martin et al., 2022; Woo, 2025). Semi-structured interviews were used to obtain rich descriptions of participants’ perspectives, emotional concerns, and expectations regarding the potential use of VR in end-of-life care. Data were analysed using inductive content analysis, allowing themes to be developed systematically from participants’ narratives rather than imposed from predefined theoretical categories.

### Study setting

This study was conducted in a public hospital in Thailand that provides both inpatient and outpatient palliative care services. The setting was selected because it reflects the integrative nature of palliative care within the Thai healthcare system, where clinical care is closely intertwined with family involvement, spiritual practices, and culturally grounded understandings of a peaceful death (National Health Commission Office, 2024).

In this context, end-of-life care often involves not only medical management but also strong participation from family members and attention to spiritual or religious needs. Family members frequently remain present at the patient’s bedside, and spiritual or religious practices such as prayer, chanting, or quiet reflection that may accompany clinical care. These sociocultural characteristics make Thai palliative care settings particularly relevant for examining how emerging technologies such as VR may be perceived within a holistic model of care that values dignity, comfort, and spiritual well-being.

Data collection was conducted between 2024 and 2025. Examining perceptions of VR within this setting allowed the researchers to explore how a digital technology might be understood in a care environment that already emphasises relational care, spiritual meaning, and community involvement at the end of life.

### Data collection methodology

To explore perceptions of the potential use of VR in end-of-life care, data were collected through semi-structured qualitative interviews with participants who had direct experience with palliative care contexts. The study aimed to gather perspectives from individuals closely involved in end-of-life care in order to obtain information-rich insights relevant to the research objectives. In qualitative research, purposive sampling is commonly used to select participants who can provide meaningful and contextually grounded accounts of the phenomenon under investigation (Patton, 2015).

Participants were recruited from a palliative care setting and were provided with both verbal and written information describing the study’s purpose, procedures, and voluntary nature prior to participation. Written informed consent was obtained from all participants before interviews were conducted. Participants were also informed that they could withdraw from the study at any time without any consequences for their medical care or professional role.

To obtain a range of perspectives, the study included three participant groups: terminally ill patients, family caregivers, and healthcare professionals involved in palliative care. Including multiple stakeholder groups allowed the study to explore emotional, relational, and clinical perspectives on the possible use of VR in end-of-life care and supported triangulation of viewpoints, which can strengthen credibility in qualitative research (Creswell & Poth, 2018).

### Participants and sampling

Participants were recruited using purposive sampling to ensure the inclusion of individuals with relevant experience related to palliative care and end-of-life contexts. A total of 15 participants were included in the study, representing three groups: (1) Terminally ill patients (*n* = 5): Participants in this group were adults diagnosed with life-limiting illnesses receiving palliative care services. Eligibility criteria included awareness of their illness status, the cognitive capacity to participate in an interview, and sufficient physical and emotional stability to engage in conversation. Their perspectives were important for understanding emotional, existential, and spiritual needs at the end of life. (2) Family caregivers (*n* = 5): Family caregivers were individuals who provided primary emotional or practical support to patients receiving palliative care. These participants offered insights into caregiving experiences and family perspectives regarding potential supportive technologies in end-of-life care. (3) Healthcare professionals (*n* = 5): This group included nurses and allied health professionals who had at least one year of experience working in palliative care. Their perspectives provided clinical and ethical insight into the potential feasibility, benefits, and challenges of implementing VR in palliative care settings.

Recruitment continued until sufficient informational depth was achieved across the three participant groups, allowing the researchers to develop a comprehensive understanding of participants’ perspectives.

### Data collection

Data were collected through semi-structured interviews conducted in Thai by the principal investigator, who had training and experience in qualitative interviewing and palliative care. Semi-structured interviews were chosen because they allow participants to describe their perspectives in their own words while enabling the researcher to explore key topics relevant to the study aims (Kallio et al., 2016).

Separate interview guides were developed for patients, family caregivers, and healthcare professionals. The guides were informed by previous literature on palliative care, spiritual care, and emerging uses of virtual reality in healthcare. The interview questions focused on participants’ emotional, existential, and spiritual needs at the end of life; their familiarity with VR; perceptions of its possible role in palliative care; anticipated benefits; potential concerns; and conditions that might influence its acceptability in clinical practice.

Participants were also encouraged to discuss any concerns, fears, or reservations they might have regarding the possible use of VR in end-of-life care. This approach allowed the researchers to capture both positive expectations and perceived risks associated with immersive technologies in sensitive care contexts.

Interviews typically lasted 45–60 minutes and were conducted in quiet, private areas within the hospital to ensure confidentiality. For patients whose medical condition limited mobility, interviews were conducted at the bedside in accordance with their comfort and clinical condition. With participants’ permission, all interviews were audio-recorded and later transcribed verbatim.

In addition to audio recordings, the researcher maintained field notes during and immediately after each interview. These notes documented contextual observations, non-verbal cues, and reflections on the interview process. Field notes can provide valuable contextual information that supports interpretation of qualitative data (Phillippi & Lauderdale, 2018).

### Characteristics of participants

A total of 15 participants took part in the study, representing three stakeholder groups involved in palliative care: terminally ill patients, family caregivers, and healthcare professionals. Including multiple groups allowed the study to capture diverse perspectives on emotional, spiritual, and practical aspects of end-of-life care and the potential role of VR in this context. The healthcare professional group (*n* = 5) consisted of nurses and allied health practitioners working in palliative care. All were female and ranged in age from 30 to 50 years, with a mean age of 38.6 years. Each participant had at least one year of clinical experience in palliative care, enabling them to provide professional insights into the feasibility, ethical considerations, and potential clinical implications of VR use in end-of-life care.

The patient and family caregiver group (*n* = 10) included five patients receiving palliative care for advanced, life-limiting illnesses and five family caregivers who were actively involved in supporting these patients. Participants in this group ranged in age from 30 to 54 years, with a mean age of approximately 43.4 years. This group included 60% male and 40% female participants. Patients shared perspectives on emotional, existential, and spiritual needs associated with advanced illness, while caregivers provided insights shaped by their experiences supporting loved ones during the end-of-life process. A summary of participant characteristics is presented in Table I.

**Table I.** Contextual characteristics of patients and family caregivers related to emotional and existential needs.

Participant ID	Age (years)	Diagnosis/Current condition	Emotional and existential stressors	Expressed needs at the end of life
P01 (Patient)	75	Cancer; unable to walk for 3 weeks	Financial burden, concern for family, loss of independence, toileting difficulties	Desire to return home, supportive equipment (e.g., electric bed), comfort
P04 (Patient)	74	Cholangiocarcinoma; chest pain	Concern about unfinished responsibilities (orchard), uncertainty about future	Relief from suffering, emotional peace, connection to meaningful places
H01 (Patient)	36	Bone cancer; severe musculoskeletal pain	Limited mobility, treatment-related financial burden, role disruption	Rest, attentive and compassionate care, emotional support
C01 (Caregiver)	40	Primary caregiver for father with metastatic cancer	Worry about home environment and pets, caregiving burden	Facilitating home-based end-of-life care, maintaining normalcy
C02 (Caregiver)	78	Primary caregiver for husband with nasopharyngeal cancer	Communication difficulties, distance from home, emotional strain	Maintaining emotional connection with family, meaningful interaction

Note: This table illustrates contextual factors shaping emotional and existential needs, including loss of independence, unfinished responsibilities, and the desire for home, comfort, and meaningful connection.

### Data analysis

All interviews were audio-recorded with participants’ permission and transcribed verbatim in Thai. Transcripts were reviewed alongside the recordings to ensure accuracy and completeness prior to analysis. The data were analysed using inductive content analysis, which allows themes and categories to be developed systematically from the data rather than imposed from predefined theoretical frameworks (Elo & Kyngäs, 2008). This analytic approach is commonly used in qualitative descriptive studies to produce a clear and comprehensive summary of participants’ perspectives.

The analysis followed several iterative steps. First, transcripts were read repeatedly to achieve familiarisation with the data. Second, meaningful segments of text relevant to the research questions were identified and coded line by line. Third, codes with conceptual similarities were grouped into subcategories and broader categories. Finally, these categories were synthesised into themes that represented shared patterns across participant groups. To strengthen analytic rigour, two members of the research team independently coded an initial subset of transcripts and then met to compare coding decisions and refine the coding framework. Discrepancies were resolved through discussion until consensus was reached. The remaining transcripts were subsequently coded using the agreed coding structure. Throughout the analytic process, analytic memos were maintained to document emerging interpretations, reflexive insights, and decisions made during coding and theme development. This process helped ensure transparency and consistency in the analysis. The analysis focused on identifying participants’ perceptions of the potential benefits, challenges, and ethical considerations associated with VR in end-of-life care, as well as the emotional and spiritual meanings associated with its anticipated use (Gaina et al., 2024; Niki et al., 2024).

### Trustworthiness

The rigour of the study was enhanced by applying established criteria for trustworthiness in qualitative research, including credibility, dependability, confirmability, and transferability (Lincoln & Guba, 1985). Credibility was supported through prolonged engagement with the data, triangulation of participant groups (patients, caregivers, and healthcare professionals), and member reflection on selected interpretations during the analytic process. Dependability was strengthened through the maintenance of a detailed audit trail documenting recruitment decisions, interview procedures, coding processes, and theme development. Confirmability was enhanced through peer debriefing and reflexive memo writing, which allowed the research team to examine analytic decisions and minimise potential researcher bias. Transferability was supported by providing thick descriptions of the study context, participants, and analytic procedures so that readers can assess the relevance of the findings to other palliative care settings (Phetcharat et al., 2023; Pitanupong & Janmanee, 2021).

### Ethical consideration

Ethical approval for the study was obtained from the [Institutional Review Board/Ethics Committee—redacted for review] prior to data collection. All participants received detailed verbal and written information explaining the purpose of the study, the procedures involved, and their rights as research participants. Written informed consent was obtained from each participant before participation. Participants were informed that their involvement was entirely voluntary and that they could withdraw from the study at any time without any impact on their medical care or professional responsibilities.

Given the sensitive nature of end-of-life discussions, particular attention was given to participants’ emotional well-being during interviews. The interviewer monitored participants for signs of emotional distress and paused or redirected the conversation if needed. Participants were reminded that they could stop the interview at any time. Confidentiality was maintained by removing identifying information from transcripts and using pseudonyms or participant codes in all study records and publications. Audio recordings and transcripts were stored securely and were accessible only to members of the research team. These procedures were implemented to protect participants’ privacy and dignity in accordance with ethical principles for research involving vulnerable populations (World Medical Association, 2013).

## Results

Through inductive content analysis, four interrelated themes were developed to explain how participants understood end-of-life needs and the potential role of VR in responding to those needs. These themes were analytically connected rather than independent. Emotional and existential needs formed the central context, while spirituality and meaning shaped how participants interpreted these needs. Facilitators and barriers influenced the perceived acceptability and feasibility of VR, and together these dimensions contributed to the broader concept of compassionate innovation in end-of-life care.

### Emotional and existential needs: confronting loss and adaptation

Most terminally ill patients described their experiences as characterised by progressive physical decline, including loss of mobility, dependence on others for basic activities, and restricted movement. These changes were often associated with reduced self-worth and disruption of previously held family and social roles. Male participants, in particular, who had previously served as primary providers, reported emotional distress related to loss of responsibility and autonomy, which was sometimes perceived as more distressing than physical pain.

As shown in Table I, participants’ experiences were influenced not only by illness severity but also by contextual stressors, such as financial concerns, unfinished responsibilities, and worry about family members. Patients expressed urgent needs related to comfort, dignity, and basic care, while family caregivers described emotional strain associated with caregiving responsibilities and separation from home.

A strong and recurring theme among most patients was the desire to return home, which symbolised emotional safety, identity, and continuity. One participant stated: *“I want to go home. I want to see my cat one more time before I die” (P01).*

Another participant expressed a longing to reconnect with meaningful life roles: *“I want to go back to my durian orchard, even though I know I probably can’t anymore” (P04).*

Participants varied in their level of emotional readiness. Those who remained concerned about unresolved responsibilities reported greater emotional distress, whereas those who had reached some level of acceptance expressed fewer emotional conflicts. These differences highlight the importance of considering individual readiness when introducing supportive interventions such as VR.

### Emotional and spiritual needs: seeking peace and connection

Across all participant groups, emotional and spiritual needs were described as central to end-of-life care, often taking precedence over physical comfort alone. Most healthcare professionals emphasised that achieving peace, acceptance, and emotional calmness was a key goal of care.

As summarised in Table II, emotional comfort was closely associated with relaxation and relief from persistent worry. Several participants suggested that exposure to calming environments, such as natural scenes, could reduce intrusive thoughts: *“If I could see a waterfall or hear nature sounds or prayers, I think I would feel calmer and stop overthinking” (P05).*

**Table II.** Themes and subthemes related to emotional and spiritual needs, with example quotations.

Theme	Subtheme	Example quotations
Emotional and existential needs	Desire to return home and maintain identity	“I want to go home, but I can’t right now. If there were glasses that let me see my house and my cat as if it were real, that would be good.” (P01)
	Need for comfort, relaxation, and relief from distress	“If I could watch waterfalls, hear nature sounds, or listen to prayers, I think I would feel calmer and stop overthinking.” (P05)
Spirituality and meaning	Religious and spiritual connection	“I want to see the monk I used to worship again, as if I were really at the temple, hearing the chanting.” (RN01)
	Acceptance, letting go, and preparation for death	“I know the time will come one day, but I want to see the things I love before I go.” (RN05)

Note: The themes demonstrate how emotional and existential needs are closely intertwined with spirituality and meaning, particularly in relation to comfort, acceptance, and preparation for death in the Thai palliative care context.

Spirituality was also identified by most participants as a significant source of meaning. In the Thai context, spiritual practices that particularly Buddhist rituals were closely linked to acceptance and preparation for death. Several participants expressed a desire to reconnect with religious experiences: *“I want to see the monk I used to worship again, as if I were really at the temple, hearing the chanting” (RN01).*

Acceptance and letting go were often associated with reassurance about loved ones and opportunities to reconnect with meaningful spiritual experiences, demonstrating the close relationship between emotional and spiritual well-being (Table III).

**Table III.** Facilitators and barriers influencing perceptions of virtual reality across participant groups.

Participant group	Perceived facilitators (potential benefits)	Perceived barriers (concerns and challenges)
Patients	Interest in VR as a means of relaxation, emotional comfort, and symbolic return to meaningful places	Visual discomfort, dizziness, weight of headset, physical limitations
Family caregivers	Perceived VR as a more immersive and meaningful way to support emotional connection compared to standard communication methods	Concern about confusion between virtual and real experiences, emotional impact
Healthcare professionals	Viewed VR as a potentially valuable tool for supporting emotional and spiritual care and enhancing patient experience	Increased workload, usability challenges among older patients, need for staff support and training

Note: Perceptions of VR reflect a balance between facilitators (emotional, spiritual, and experiential benefits) and barriers (physical, emotional, and practical constraints), corresponding to the theme of *facilitators and barriers* influencing anticipated acceptability and feasibility.

### Perceptions of virtual reality: from novelty to conditional acceptance

Participants’ perceptions of VR varied across groups. Most patients had no prior experience with VR, but expressed interest when it was described as a tool for relaxation and emotional comfort. However, several participants raised concerns regarding visual discomfort, dizziness, and the physical weight of VR headsets.

Family caregivers generally expressed positive attitudes, viewing VR as a more immersive alternative to conventional communication methods. One caregiver stated: *“VR feels more like actually being there with family than talking on LINE” (C02).*

Healthcare professionals also viewed VR as a potentially valuable addition to emotional and spiritual care. However, many identified practical concerns, including time constraints, staff workload, and usability among older patients. These findings reflect a tension between technological potential and practical implementation in clinical settings.

### Opportunities for VR content development in end-of-life care

Participants identified several types of VR content that could be meaningful in end-of-life care. Four main categories were consistently described: *familiar environments (e.g., home, personal spaces), nature-based settings (e.g., beaches, waterfalls), spiritual and religious environments (e.g., temples, chanting), and meaningful or “last wish” experiences.* Most participants emphasised that personalisation was essential. Recreating familiar environments, such as one’s home or interactions with pets, was perceived as particularly meaningful, supporting emotional comfort and continuity of identity.

Nature-based environments were associated with calmness and stress reduction, while spiritual content supported peace and acceptance. VR was also seen as a way to symbolically fulfil meaningful experiences that were no longer physically possible.

### Barriers to VR use: physical, emotional, and practical constraints

Despite general interest in VR, participants identified several barriers. *Physical limitations,* including fatigue, pain, delirium, and visual impairment, were commonly reported among patients. *Emotional risks* were also identified. Several participants expressed concern that immersive experiences might increase sadness or longing, particularly when viewing representations of home or loved ones. *Technical and practical challenges* were emphasised by healthcare professionals, including the need for staff assistance, setup time, and reliable infrastructure. One nurse explained: *“Older patients may think VR is too difficult, but if someone helps them put it on, they are willing to try” (H01).*

These findings highlight the importance of guided use, patient selection, and clinical feasibility.

### Integrative synthesis: from comfort to meaning

Across all themes, participants emphasised three key considerations for integrating VR into end-of-life care: *personalisation of content*, *multi-sensory engagement, and holistic evaluation of outcomes.* Participants highlighted that outcomes should not be limited to symptom relief but should include emotional comfort, dignity, peace, and meaning.

Overall, VR was perceived as a potentially compassionate technology not only as a tool for distraction, but as a means of supporting emotional expression, spiritual connection, and a sense of autonomy during advanced illness.

## Discussion

This study explored how terminally ill patients, family caregivers, and healthcare professionals perceived the potential use of VR in end-of-life care. Using inductive content analysis, four interrelated themes were developed: emotional and existential needs, spirituality and meaning, facilitators and barriers, and compassionate innovation. These themes are analytically connected, with emotional and existential needs forming the central context, while spirituality and meaning shape interpretation, and facilitators and barriers influence perceived feasibility. The findings should be interpreted as reflecting anticipated perceptions and acceptability of VR, rather than direct clinical outcomes.

### Emotional and existential needs: beyond symptom control

The findings demonstrate that participants’ experiences at the end of life are shaped not only by physical symptoms, but also by emotional and existential concerns, including loss of independence, disrupted identity, and concern for unfinished responsibilities. The strong desire to “return home,” expressed by many participants, reflects a need for emotional security, familiarity, and continuity of identity, rather than a purely practical request. This interpretation is consistent with previous research showing that existential distress such as loss of meaning, identity disruption, and concerns about family often contributes significantly to suffering at the end of life (Phetcharat et al., 2023; Pitanupong & Janmanee, 2021). In this study, participants suggested that VR could potentially support these needs by enabling symbolic return to meaningful places, thereby restoring a sense of connection and continuity when physical mobility is limited.

Importantly, the findings also highlight variability in emotional readiness among participants. Some individuals expressed unresolved concerns, while others demonstrated greater acceptance. This suggests that the introduction of VR should be individualised and sensitive to emotional state, consistent with recommendations for patient-centred palliative care (Woo, 2025).

### Spirituality and meaning: culturally grounded interpretations of VR

Spirituality emerged as a central component of end-of-life experience, particularly within the Thai context, where Buddhist beliefs and practices shape understandings of acceptance, merit-making, and preparation for death (National Health Commission Office, 2024). Participants described VR as having the potential to facilitate spiritual engagement, such as experiencing temple environments or listening to chanting, even when physical participation is not possible. These findings are consistent with international studies suggesting that immersive environments can support reflection, emotional calmness, and spiritual well-being (Moscato et al., 2021). However, this study extends previous work by demonstrating that such experiences are culturally embedded and symbolically meaningful, rather than universally interpreted.

The subtheme of acceptance and letting go further highlights the role of spirituality in achieving emotional resolution. Participants associated meaningful experiences such as reconnecting with loved ones or engaging in familiar rituals with a sense of peace and readiness. This aligns with holistic palliative care frameworks, which emphasise the integration of emotional, spiritual, and relational dimensions of care. In this context, VR may be understood not only as a technological tool, but as a potential medium for meaning-making and spiritual continuity, particularly when adapted to cultural and individual preferences.

## Facilitators and barriers: contextualising VR in clinical practice

While participants generally expressed interest in VR, they also identified several barriers related to physical, emotional, and practical factors. Patients reported concerns about discomfort, such as dizziness or fatigue, while caregivers expressed concern about potential emotional confusion. Healthcare professionals highlighted issues related to workload, time constraints, and usability, particularly for older patients. These findings are consistent with previous research demonstrating that the implementation of VR in healthcare settings requires careful consideration of both benefits and limitations (Pohan et al., 2024). Importantly, participants emphasised that VR should not replace human interaction but rather serve as a complementary intervention within a relational care framework.

This reflects a broader tension between technological innovation and person-centred care, which is particularly relevant in palliative settings. Consistent with Thai nursing literature, participants emphasised the continued importance of human presence, empathy, and attentiveness in care delivery (Chatchumni et al., 2016; Chatchumni et al., 2015). The concept of assisted use, where healthcare professionals support patients during VR experiences, emerged as a key strategy to address these concerns. This aligns with recommendations for ethically responsible implementation of immersive technologies (Woo, 2025).

### Toward compassionate innovation: from experience to meaning

A key contribution of this study lies in reframing VR as a form of compassionate innovation, in which technology is integrated into care in a way that supports emotional, spiritual, and existential well-being. Participants emphasised that meaningful VR experiences should be: personalised to individual histories and preferences, multi-sensory, incorporating visual, auditory, and symbolic elements, and culturally relevant, reflecting spiritual beliefs and practices.

These findings are consistent with emerging literature advocating patient-centred and culturally sensitive design of digital health technologies (Maheta et al., 2025; Niki et al., 2024). Importantly, participants highlighted that outcomes should extend beyond symptom reduction to include peace, dignity, emotional comfort, and meaning. This challenges conventional biomedical evaluation frameworks and supports broader definitions of well-being in palliative care. Taken together, the findings suggest that VR may have potential as a supportive intervention that bridges physical limitation and experiential meaning. However, implementation should be approached cautiously, with attention to individual readiness, cultural context, and ethical considerations.

These findings provide a contextually grounded foundation for future research and development of VR interventions that are ethically sensitive, culturally appropriate, and aligned with holistic palliative care principles.

## Strengths and limitations

This study has several important strengths. First, it incorporates triangulated perspectives from terminally ill patients, family caregivers, and healthcare professionals, allowing for a more comprehensive understanding of end-of-life needs and the perceived role of VR. This multi-stakeholder approach enhances the credibility and depth of the findings (Creswell & Poth, 2018). Second, the study is situated within a Thai sociocultural context, where family relationships, Buddhist beliefs, and culturally embedded understandings of a peaceful death play a central role. This context-specific focus provides valuable insights that extend beyond predominantly Western literature on VR in palliative care. Third, the study contributes conceptually by framing VR as a potentially compassionate and meaning-oriented technology, rather than solely as a tool for symptom management, thereby broadening current discussions in digital health and palliative care (Pohan et al., 2024).

However, several limitations should be acknowledged. First, the study was conducted in a single healthcare setting with a relatively small purposive sample, which may limit the transferability of findings to other contexts. Second, the findings are based on anticipated perceptions of VR rather than direct experience, as participants did not engage with VR during the study. Therefore, the results should not be interpreted as evidence of clinical effectiveness or actual patient outcomes. Third, as an interpretive qualitative descriptive study, the findings are contextually grounded and influenced by participants’ sociocultural backgrounds, as well as the researchers’ interpretive processes. Although strategies such as reflexivity, peer debriefing, and audit trails were used to enhance rigour, some degree of interpretive subjectivity is inherent in qualitative research (Lincoln & Guba, 1985). Finally, participants’ responses may have been influenced by their health status or emotional condition, particularly among patients in advanced stages of illness.

## Implications and future research

The findings of this study have important implications for clinical practice, research, and education. For practice, the results suggest that any future implementation of VR in palliative care should be individualised, culturally sensitive, and guided by careful assessment of emotional readiness and spiritual preferences. VR should be used as a complementary tool, rather than a replacement for human interaction, and should be facilitated by healthcare professionals who can support patients throughout the experience.

For research, future studies should move beyond anticipated perceptions to examine the actual effects of VR interventions on emotional, spiritual, and clinical outcomes. Experimental or mixed-methods designs could be used to evaluate feasibility, acceptability, and potential benefits over time. In addition, there is a need to develop and test culturally adapted VR content, particularly in non-Western contexts where meanings of spirituality, family involvement, and end-of-life experiences may differ (Maheta et al., 2025; Niki et al., 2024). Future research should also explore ethical considerations, including emotional risks such as distress, overstimulation, or intensified longing, as well as issues related to consent and patient vulnerability.

In terms of education, healthcare professionals may require training to assess both the therapeutic potential and ethical implications of immersive technologies in palliative care settings. Interdisciplinary collaboration between clinicians, technologists, and cultural experts will be essential to ensure that VR interventions are safe, meaningful, and contextually appropriate.

## Conclusion

This qualitative descriptive study provides a contextually grounded understanding of how VR is perceived as a potential supportive technology in end-of-life care within a Thai setting. The findings indicate that VR may be understood not only as a tool for distraction, but as a medium with potential to support emotional comfort, symbolic connection, spiritual reflection, and meaning making, particularly when physical limitations restrict real-world experiences. Importantly, participants emphasised that the value of VR lies not only in its technical capabilities, but in its ability to align with human needs for dignity, peace, and relational connection at the end of life. At the same time, concerns related to physical comfort, emotional impact, and practical implementation highlight the need for careful, ethically informed integration of such technologies.

These findings should be interpreted cautiously, as they reflect anticipated perceptions rather than direct intervention outcomes. Nevertheless, they offer important insights into how VR may be meaningfully incorporated into palliative care when guided by cultural understanding, patient readiness, and clinical judgement. Rather than positioning VR as a technological solution, this study suggests that its potential lies in supporting a model of compassionate innovation, in which technology enhances rather than replaces human-centred care. Future research is needed to evaluate how such approaches can be implemented in practice while maintaining sensitivity to the emotional, spiritual, and cultural dimensions of end-of-life care.

## Supplementary Material

Supplementary Material Author contributions.pdf

## Data Availability

The qualitative data generated and analysed during this study are not publicly available due to the sensitive nature of the data and the need to protect participant confidentiality. De-identified data may be available from the corresponding author upon reasonable request, subject to ethical approval and applicable data protection regulations.
