# Supplementary material for: Between reality and meaning: a qualitative study on virtual reality as a compassionate technology in end-of-life care
Source: Int J Qual Stud Health Well-being. 2026 May 6;21(1):2669695. doi: 10.1080/17482631.2026.2669695 (PMC13159595; doi:10.1080/17482631.2026.2669695)
Supplement: Supplementary Material — Author contributions.pdf [file ZQHW_A_2669695_SM0852.pdf]

Author contributions

Use this form to specify the contribution of each author of your manuscript. A distinction is made between five types of contributions: Conceived and designed the analysis; Collected the data; Contributed data or analysis tools; Performed the analysis; Wrote the paper.

For each author of your manuscript, please indicate the types of contributions the author has made. An author may have made more than one type of contribution. Optionally, for each contribution type, you may specify the contribution of an author in more detail by providing a one-sentence statement in which the contribution is summarized. In the case of an author who contributed to performing the analysis, the author's contribution for instance could be specified in more detail as 'Performed the computer simulations', 'Performed the statistical analysis', or 'Performed the text mining analysis'.

If an author has made a contribution that is not covered by the five pre-defined contribution types, then please choose 'Other contribution' and provide a one-sentence statement summarizing the author's contribution.

Manuscript title: Between Reality and Meaning: A Qualitative Study on Virtual Reality as a Compassionate Technology in End-of-Life Care

Author 1: Assoc. Prof. Dr. Manaporn Chatchumni

☒ Conceived and designed the analysis
Specify contribution in more detail (optional; no more than one sentence)

☒ Collected the data
Specify contribution in more detail (optional; no more than one sentence)

☒ Contributed data or analysis tools
Specify contribution in more detail (optional; no more than one sentence)

☒ Performed the analysis
Specify contribution in more detail (optional; no more than one sentence)

☒ Wrote the paper
Specify contribution in more detail (optional; no more than one sentence)

☒ Other contribution
Specify contribution in more detail (required; no more than one sentence)

Author 2: Mr.Rawiphon Chotikunnan
Author 6 : Assoc. Prof. Phichitphon Chotikunnan

☒ Conceived and designed the analysis
Specify contribution in more detail (optional; no more than one sentence)

☐ Collected the data
Specify contribution in more detail (optional; no more than one sentence)

☐ Contributed data or analysis tools
Specify contribution in more detail (optional; no more than one sentence)

☒ Performed the analysis
Specify contribution in more detail (optional; no more than one sentence)

☒ Wrote the paper
Specify contribution in more detail (optional; no more than one sentence)

☒ Other contribution
Specify contribution in more detail (required; no more than one sentence)

Author 3: Mr.Ravinan Thatsiriniratkul
Author 4: Mr.Thongdech Prasertsri
Author 5: Ms.Petpailin Phibunnithikasem

☐ Conceived and designed the analysis
Specify contribution in more detail (optional; no more than one sentence)

☐ Collected the data
Specify contribution in more detail (optional; no more than one sentence)

☒ Contributed data or analysis tools
Specify contribution in more detail (optional; no more than one sentence)

☒ Performed the analysis
Specify contribution in more detail (optional; no more than one sentence)

☒ Wrote the paper
Specify contribution in more detail (optional; no more than one sentence)

☒ Other contribution
Specify contribution in more detail (required; no more than one sentence)
